# Causal Relationships Between Immune Cell Traits, Plasma Metabolites, and Asthma: A Two‐Step, Two‐Sample Mendelian Randomization Study

**DOI:** 10.1111/crj.70097

**Published:** 2025-06-23

**Authors:** Zhuozheng Hu, Peihao Xu, Jiajun Wu, Weijun Zhou, Yajie Zhou, Lei Xie, Wenxiong Zhang, Yong Cheng

**Affiliations:** ^1^ Department of Thoracic Surgery The Second Affiliated Hospital, Jiangxi Medical College, Nanchang University Nanchang China; ^2^ School of Stomatology, Jiangxi Medical College Nanchang University Nanchang China; ^3^ Department Respiratory and Critical Care Medicine The Second Affiliated Hospital, Jiangxi Medical College, Nanchang University Nanchang China

**Keywords:** asthma, immune cell, Mendelian randomization, plasma metabolites

## Abstract

**Background:**

Considerable evidence suggests a strong link between immune cell traits (ICTs) and asthma development via plasma metabolites (PMs), but the causality between ICTs and asthma is still unclear, mainly due to issues like selection bias. Our research was designed to investigate the causality between ICTs, PMs, and asthma and to provide a clearer explanation of these relationships.

**Methods:**

Utilizing the GWAS database, this study employed a two‐step, two‐sample Mendelian randomization (MR) approach and the inverse variance weighted (IVW) method to investigate the causality between ICTs and asthma, as well as between PMs and asthma. Lastly, we calculated the mediated proportion of PMs as mediators in the link between ICTs and asthma.

**Results:**

Excluding heterogeneity and pleiotropy, MR analysis identified 13 ICTs (CD14 on CD33br HLA DR+ CD14dim, etc.) and asthma causality, and no reverse causality was observed. In addition, 27 PMs (androsterone sulfate levels, succinate levels, etc.) were also causally associated with asthma. Mediate MR indicated −9.81% (−1.2%, −18.4%) of the effect of CD24 on IgD+ CD38br on asthma is mediated by S‐methylcysteine sulfoxide levels, with a mediated effect value (*p* = 0.006) is 0.003 (0.0004, 0.006); 21.4% (6.2%, −36.6%) of the effect of CD3 on CD28+ CD4+ on asthma is mediated by 1‐myristoyl‐2‐arachidonoyl‐GPC (14:0/20:4) levels, with a mediated effect value (*p* = 0.025) is 0.004 (0.001, 0.007).

**Conclusions:**

We identified two pathways by which ICTs can impact asthma through PMs, which might help in identifying potential targets for personalized treatment approaches.

## Graphical abstract

Fourteen ICTs were found to be causal relationship with asthma, and there are 27 PMs that can influence asthma. We found that ICTs (CD24 on IgD+ CD38br and CD3 on CD28+ CD4+) influence asthma through two pathways, mediated by PMs (S‐methylcysteine sulfoxide levels and 1‐myristoyl‐2‐arachidonoyl‐GPC [14:0/20:4] levels).

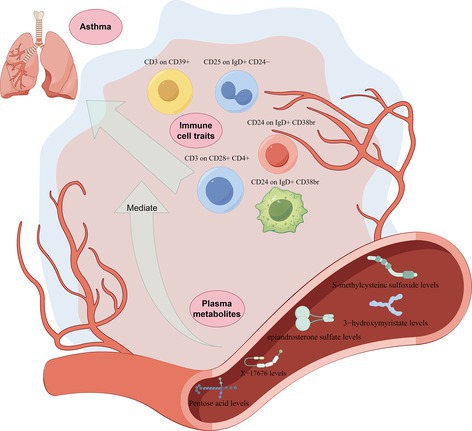

**Abbreviations**
3‐CMPFP: 3‐carboxy‐4‐methyl‐5‐pentyl‐2‐furanpropionate
FWER: family‐wise error rate
ICLs: immune cell traits
IVs: instrumental variables
IVW: inverse variance weighting
KEGG: Kyoto Encyclopedia of Genes and Genomes
LOO: leave‐one‐out
MDSC: myeloid‐derived suppressor cell
MR: Mendelian randomization
MR‐PRESSO: Mendelian randomization pleiotropy residual sum and outlier
OR: odds ratio
PMs: plasma metabolites
SAH: S‐adenosylhomocysteine
SMPDB: Small Molecule Pathway Database
SNP: single nucleotide polymorphism
TBNK: B cells, natural killer cells, and T cells

## 1 Introduction

Globally affecting about 334 million people of all ages, asthma ranks as a major chronic respiratory disease, contributing significantly to health issues, mortality rates, and economic expenses [1]. Asthma development is driven by a multifaceted interaction of genetic, environmental, infectious, and nutritional elements, with genetics having a particularly prominent impact [2]. The lack of understanding regarding asthma mechanisms leads to current treatments not being uniformly effective. Consequently, a deeper insight into the pathogenesis of asthma is critical for designing targeted interventions and improving how the disease is managed.

Asthma is a chronic condition involving inflammation of the airways, where ICTs infiltrate and become activated, causing the airways to constrict and narrow. These conditions produce symptoms such as difficulty in breathing, wheezing, and tightness in the chest. ICTs are therefore central to the pathogenesis and physiological processes of asthma [3]. Metabolomics has revealed new biomarkers related to the condition by analyzing small molecules like carbohydrates, amino acids, organic acids, nucleotides and lipids [4]. Research shows that more and more PMs are being found to be associated with asthma, but the features of asthma are mainly associated with aberrant immune responses [5]. However, no research has yet integrated ICTs and PMs to identify novel biomarkers associated with asthma.

MR stands as the prevailing causal analysis technique in clinical research, exploiting genetic variations linked to exposures or risk factors to gauge potential causal associations with outcomes [6]. MR frequently relies on single‐nucleotide polymorphisms (SNPs) identified via genome‐wide association studies (GWASs) to serve as genetic variations and instrumental variables (IVs). According to the principle of random allelic allocation, SNPs are randomly assigned to the next generation, so MR can minimize the intervention of confounding factors and reverse causation, so as to make the study more reliable [7].

In our study, asthma related data were downloaded from GWAS aggregated statistics as the outcome, immune cell related data were obtained from existing literature as the exposure factor and PMs were used as the mediator [8]. The causal relationship between them was determined by MR analysis [9], in order to explore the unknown pathogenesis of asthma and new treatment prospects.

## 2 Materials and Methods

### 2.1 Data Sources

**FIGURE 1:**
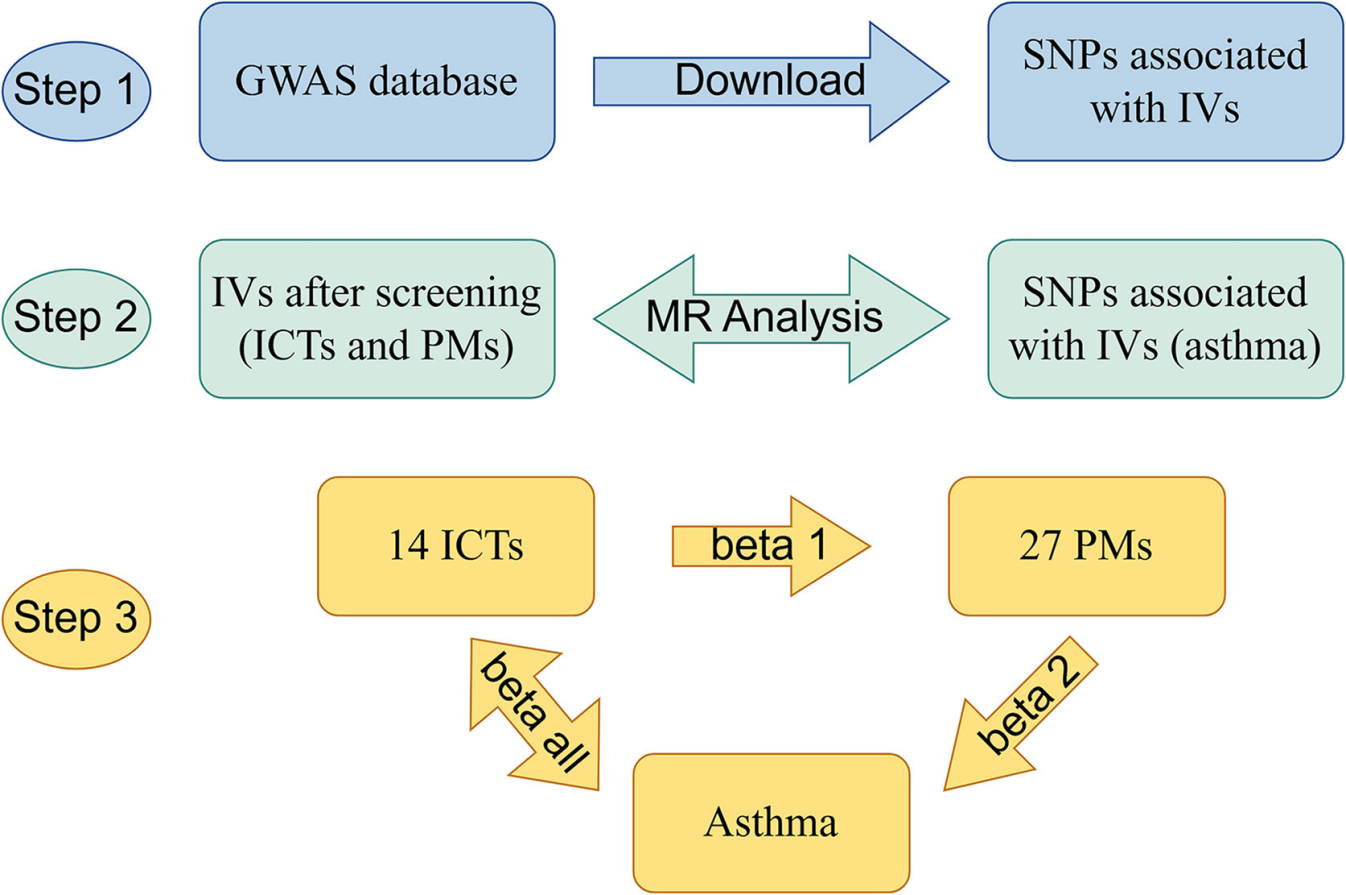
This study's design and flowchart.

The flowchart is depicted in Figure 1. The data for 731 ICTs (Ebi‐a‐GCST0001391 to Ebi‐a‐GCST0002121) were sourced from the GWAS catalog (https://gwas.mrcieu.ac.uk/). These ICTs include seven categories: B cells, CDCs, mature T cell stages, monocytes, myeloid cells, TBNK (B cells, natural killer cells, and T cells), and treg groups. We employed summary statistics from a GWAS on psychiatric disorders, using data from a cohort of 7824 individuals of European descent sourced from the GWAS database (accession numbers for European GWASs: GCST90199621‐90201020) [10]. Data on asthma as an outcome factor were selected from the GWAS database (GCST90018795), which included a total sample of 449 500 European patients with asthma and 411 131 controls [11].

### 2.2 IVs Selection

Initially, we chose available IVs by applying a filtration criterion of *p* < 1 × 10^−5^ to the SNPs for both ICTs and PMs [12]. The clustering distance was 10 000 kb, setting the linkage disequilibrium threshold for clustering to be *r* ^2^ < 0.001. The filter condition of SNPs in asthma was set as *p* < 5 × 10^−8^. We used the *F*‐statistic to exclude weak SNPs across the 731 ICTs. The *F*‐statistic, computed as the ratio of the effect size (β) to the square of the standard error, is considered when *F* < 10. Finally, the PhenoScanner database was used to detect whether confounding factors existed in existing SNPs [13].

### 2.3 Statistical Analysis

Firstly, we conducted two‐step, two‐sample MR analyses for both ICLs and outcome factors, as well as for PMs and outcome factors. Among the five MR analysis methods we used, “MR‐Egger” [14], “Weighted median” [15], “IVW” [16], “Simple mode” [17], and “Weighted mode” [14], the IVW leverages the advantages of IVs to effectively control for confounding factors, thereby improving the accuracy and reliability of causal inference. Therefore, it is our primary analytical method. A *p*‐value < 0.001 is considered to indicate a significant causal relationship. Additionally, we performed Cochran's Q test and the MR‐Egger intercept test to examine the pleiotropy of the selected IVs (*p* > 0.05). The MR‐Egger intercept test, as well as the MR pleiotropy residual sum and outlier (MR‐PRESSO) method for detecting and correcting for heterogeneity (Q > 0.05), were used to assess heterogeneity, with MR‐PRESSO also employed to adjust for pleiotropy after removing outliers. To assess whether a specific genetic locus influences random estimates, the leave‐one‐out (LOO) sensitivity analysis was employed. Finally, variables supported by fewer than five SNPs will be excluded from the analysis. The Bonferroni correction adjusts the significance threshold for each individual test in order to control the family‐wise error rate (FWER). By applying this correction, we account for the increased likelihood of false positives when performing multiple comparisons. Specifically, the significance level for each test is divided by the number of tests conducted, making the threshold more stringent. This method ensures that the overall probability of committing at least one Type I error across all tests remains below a pre‐specified level, typically 0.05. While this approach helps to maintain the integrity of the results by minimizing false discoveries, it may also reduce the power to detect true associations, particularly in studies with many tests or small effect sizes. Therefore, it is important to interpret results with caution, acknowledging that some tests may fail to reach the significance threshold despite having potential biological relevance [18]. Kyoto Encyclopedia of Genes and Genomes (KEGG) and Small Molecule Pathway Database (SMPDB) enrichment analyses were conducted by using MetaboAnalyst 5.0.

### 2.4 Mediation Analysis

Mediation analysis is a statistical technique used to examine whether a variable acts as an intermediary in the relationship between two other variables. By applying mediation MR, we identified the pathways from ICTs through PMs to asthma, providing insights into the mechanisms by which ICTs might facilitate the onset of asthma. First, we conducted mediation analysis to evaluate the possible link between MR‐identified ICTs and PMs. Subsequently, we assessed the “indirect” effect of ICTs on asthma via PMs using a two‐step MR process. The calculation formulas were as follows: The mediated proportion = β (Mediated effect) / β (Total effect); β (Mediated effect) = β (Direct effect A) × β (Direct effect B); Total effect: The causal role of ICTs on asthma; Direct effect A: The causal role of ICTs on PMs; Direct effect B: The causal role of PMs on asthma [19].

## 3 Results

### 3.1 Instrument Variables Included in Analysis

Following the outlined methods and criteria, the IVs for ICTs and PMs were extracted. With *F*‐statistics greater than 10 for all IVs, there was no indication of weak instrument bias. Comprehensive data for these IVs are demonstrated in Table 1.

**TABLE 1:** Causal associations between immune cell traits and asthma by using the IVW method.

Exposure	Nsnp	B	Se	OR	OR_LCI95	OR_UCI95	*p*‐value
CD39+ activated Treg AC	30	−0.015	0.005	0.985	0.974	0.996	0.006
CD3‐ lymphocyte AC	18	−0.037	0.013	0.963	0.938	0.988	0.005
CD28‐ DN (CD4‐CD8‐) AC	32	−0.036	0.014	0.964	0.939	0.991	0.008
BAFF‐R on IgD+ CD38‐ unsw mem	21	0.019	0.006	1.019	1.006	1.032	0.004
BAFF‐R on IgD+ CD38br	27	0.019	0.007	1.019	1.005	1.032	0.006
BAFF‐R on transitional	24	0.020	0.008	1.020	1.005	1.035	0.008
CD19 on IgD+ CD38‐ naive	19	−0.019	0.007	0.981	0.967	0.995	0.008
CD24 on IgD+ CD38br	25	−0.020	0.008	0.980	0.966	0.995	0.009
CD25 on IgD+ CD24—	26	0.017	0.005	1.017	1.006	1.028	0.002
CD3 on CD39+ secreting Treg	28	−0.032	0.009	0.968	0.951	0.985	3.26E‐04
CD3 on CD28+ CD4+	25	−0.033	0.011	0.967	0.946	0.989	0.004
CD14 on CD33br HLA DR+ CD14dim	19	0.028	0.010	1.029	1.008	1.050	0.006
HLA DR on CD33br HLA DR+ CD14—	25	0.015	0.005	1.015	1.004	1.026	0.006

Abbreviations: B, beta‐value; IVW, inverse‐variance weighted; LCI, lower confidence interval; Nsnp, number of SNPs; OR, odds ratio; Se, standard error; Snp, single nucleotide polymorphism; UCI, upper confidence interval.

### 3.2 Causal Effects of ICTs on Asthma

**FIGURE 2:**
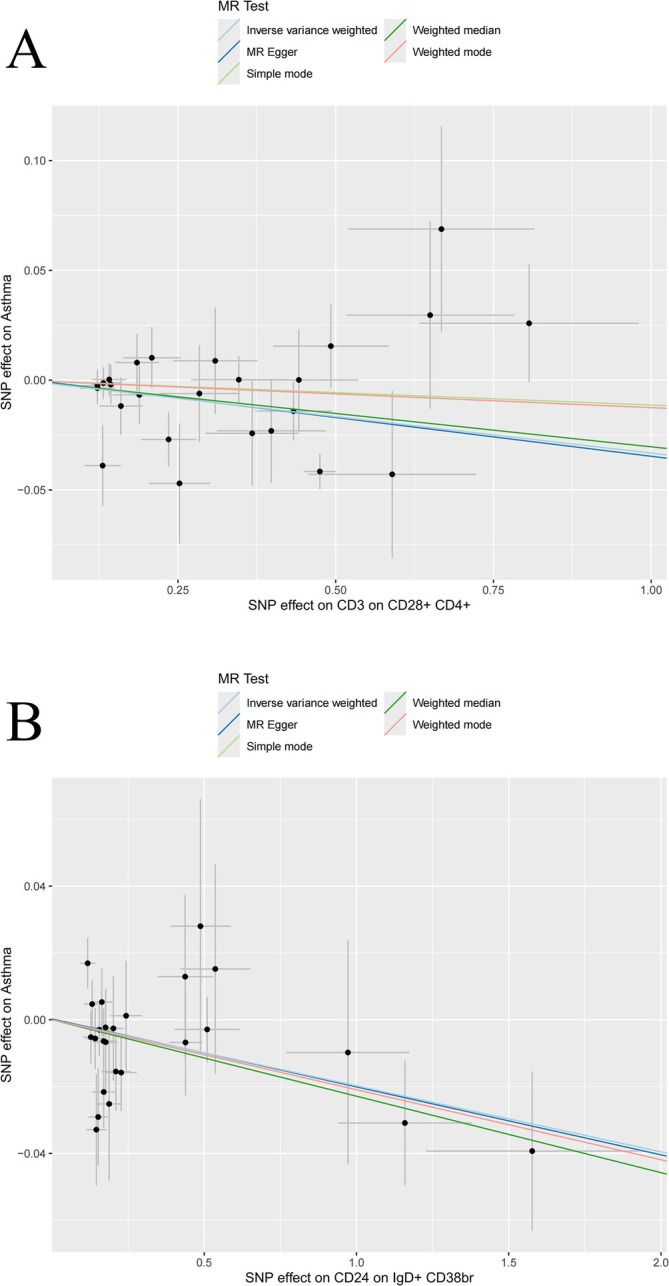
Scatter plots for the causal association between immune cell traits and asthma.

As shown in Table 1, the IVW analysis results indicate that the following six ICTs may contribute to an increased risk of asthma: CD14 on CD33br HLA DR+ CD14dim (OR = 1.03 [1.01, 1.05], *p* = 0.0064), etc. The seven ICTs also associated with a reduced risk of asthma include CD24 on IgD+ CD38br (OR = 0.98 [0.97, 1.00], *p* = 0.0090), CD3 on CD39+ secreting treg (OR = 0.97 [0.95, 0.99], *p* = 0.0003), etc. We performed multiple hypothesis testing for various ICTs and applied Bonferroni correction to adjust the significance threshold; the correction process revealed that only two ICTs remained significant (Table S1). The causality between ICTs and asthma was represented visually using scatter plots (Figures 2 and S1). Possibly due to the use of different research methods, CD28‐DN (CD4‐CD8‐) AC exhibited heterogeneity; the other ICTs did not demonstrate any heterogeneity or pleiotropy (Tables S2 and S3). The LOO analysis revealed that omitting any individual SNPs did not affect the causal estimates (Figure S2).

### 3.3 Reverse MR Analysis

We used the same five MR methods in this step to perform reverse MR analysis. After validation, the *p*‐values for all five methods were greater than 0.05, indicating that there is no reverse causation between 13 ICTs and the outcome factors (Table S4).

### 3.4 Causal Effects of PMs on Asthma

**FIGURE 3:**
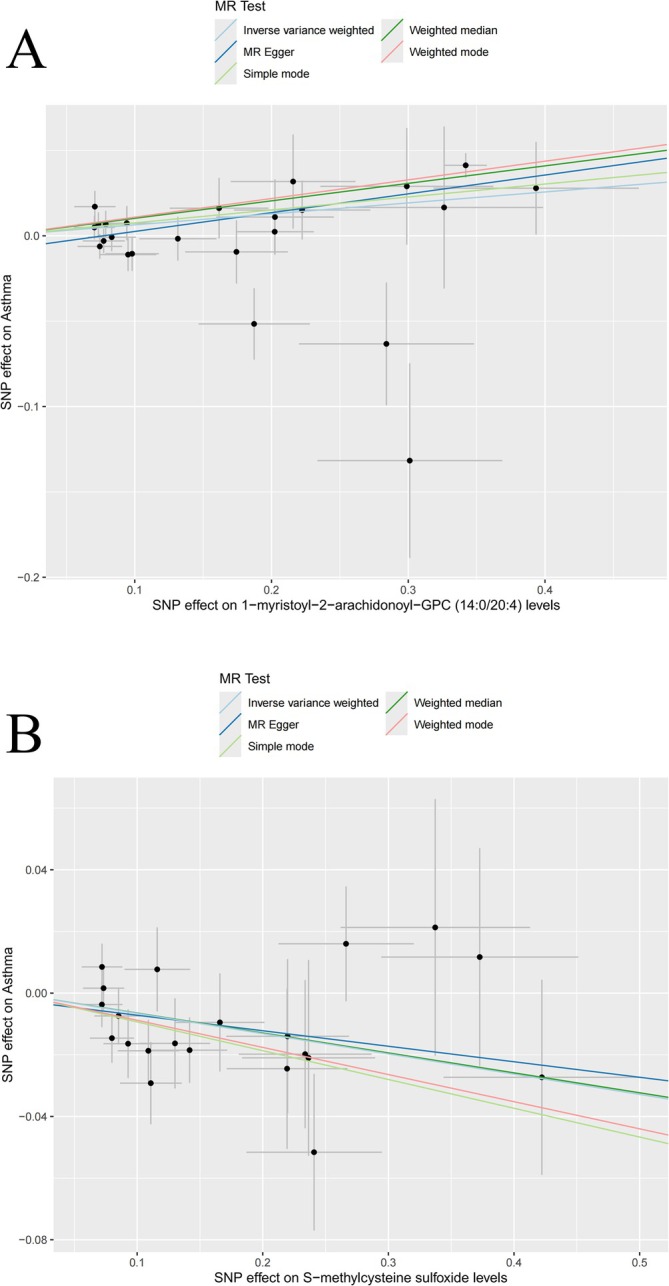
Scatter plots for the causal association between plasma metabolites and asthma.

**FIGURE 4:**
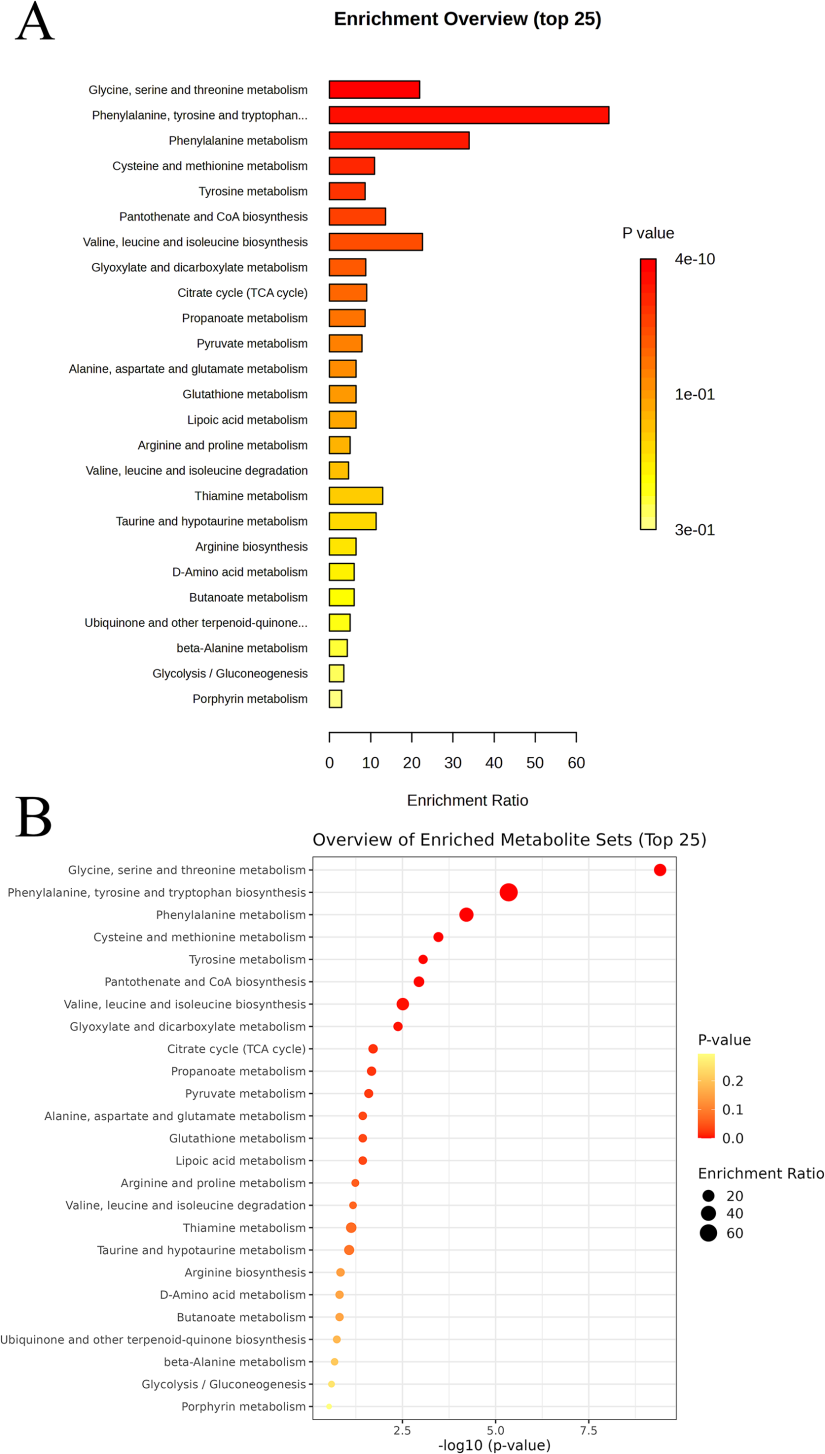
Enrichment analysis results of the causal plasma metabolites of asthma based on the Kyoto Encyclopedia of Genes and Genomes database (A, B).

**FIGURE 5:**
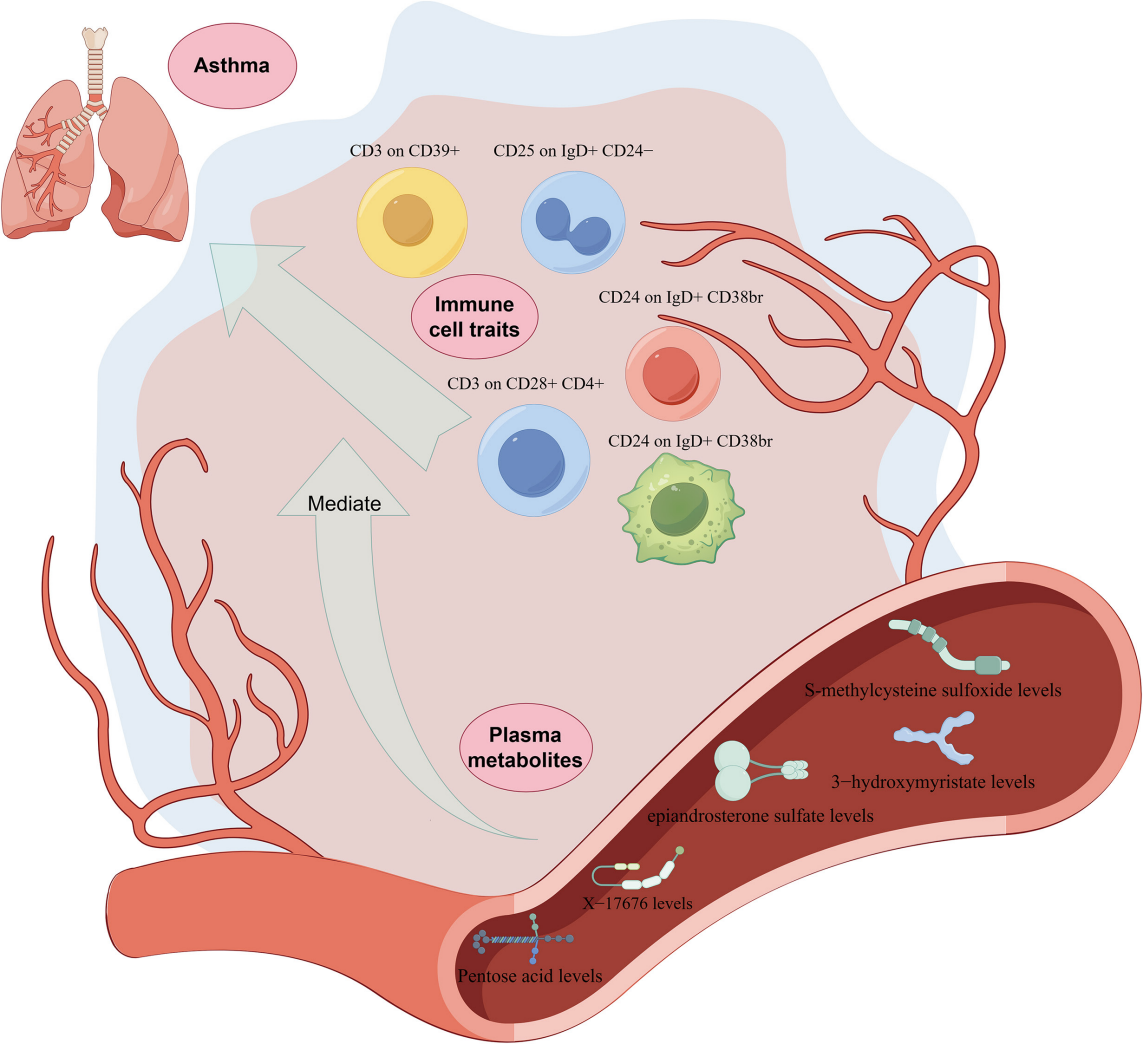
Mediation effect relationship of immune cell traits and plasma metabolites on asthma.

We used the IVW method to analyze 1400 PMs, and obtained 27 PMs that may have a causal relationship with asthma (Table 2). We conducted multiple hypothesis tests and adjusted the *p*‐values of all metabolites using the Bonferroni correction method to control the false detection rate caused by multiple comparisons. The corrected significance threshold was set at 0.00185 (i.e., 0.05/27, where 27 is the total number of metabolites tested); three PMs all passed the significance test after Bonferroni correction (Table S5). The risk of asthma may be elevated by the following 16 PMs, S‐adenosylhomocysteine (SAH) to leucine ratio, pentose acid levels, etc. The remaining 11 PMs may be protective factors for asthma, including S‐methylcysteine sulfoxide levels and X‐21364 levels. The causal relationship between PMs and asthma is shown by scatter plots (Figures 3, S3, and S4). Cochran's Q statistic‐based analysis revealed that seven PMs (e.g., N,N,N‐trimethyl‐5‐aminovalerate, succinate levels) showed significant heterogeneity (*p* < 0.05) (Table S6); this result may also be due to differences in research methods. The lack of statistical significance in both the MR‐Egger intercept test and MR‐PRESSO results implies that horizontal pleiotropy is unlikely (Table S7). The LOO analysis indicated that omitting a specific SNP did not affect the causal estimates (Figures S5 and S6). We conducted KEGG and SMPDB enrichment analyses on a set of 27 PMs. Significant enrichment in glycine, serine, and threonine metabolism was identified through KEGG analysis (Figure 4), while SMPDB analysis highlighted notable enrichment in glycine and serine metabolism (Figure S7). The mechanism of this study is illustrated in Figure 5.

**TABLE 2:** Causal associations between plasma metabolites and asthma by using the IVW method.

Exposure	Nsnp	B	Se	OR	OR_LCI95	OR_UCI95	*p*‐value
Stearidonate (18:4n3) levels	26	0.08	0.025	1.082	1.030	1.136	0.002
1‐Linoleoyl‐gpc (18:2) levels	30	−0.06	0.022	0.942	0.902	0.983	0.007
Epiandrosterone sulfate levels	23	0.05	0.015	1.046	1.015	1.078	0.003
Beta‐hydroxyisovaleroylcarnitine levels	36	0.06	0.015	1.057	1.027	1.089	1.99E‐04
Alpha‐hydroxycaproate levels	20	0.05	0.018	1.046	1.011	1.083	0.010
1‐Palmitoyl‐2‐linoleoyl‐GPE (16:0/18:2) levels	25	−0.06	0.015	0.939	0.912	0.967	2.33E‐05
5alpha‐androstan‐3beta,17alpha‐diol disulfate levels	24	0.06	0.016	1.058	1.024	1.092	6.41E‐04
5AAA,17beta‐diol monosulfate (1) levels	21	0.03	0.011	1.034	1.011	1.057	0.004
S‐methylcysteine sulfoxide levels	21	−0.07	0.022	0.936	0.897	0.977	0.003
1,2‐Dilinoleoyl‐GPC (18:2/18:2) levels	17	−0.08	0.023	0.921	0.879	0.964	3.9E‐04
1‐Stearoyl‐2‐linoleoyl‐GPE (18:0/18:2) levels	30	−0.06	0.019	0.946	0.911	0.982	0.004
1‐Myristoyl‐2‐arachidonoyl‐GPC (14:0/20:4) levels	24	0.06	0.020	1.066	1.025	1.110	0.002
1‐Oleoyl‐2‐linoleoyl‐GPE (18:1/18:2) levels	32	−0.06	0.015	0.942	0.916	0.970	4.67E‐05
N, N, N‐trimethyl‐5‐aminovalerate levels	33	0.05	0.018	1.052	1.016	1.090	0.005
3‐CMPFP levels	27	0.05	0.018	1.048	1.012	1.085	0.009
2‐Naphthol sulfate levels	23	−0.06	0.020	0.942	0.906	0.980	0.003
Pentose acid levels	22	0.06	0.020	1.057	1.015	1.100	0.007
1‐Palmitoyl‐2‐linoleoyl‐gpc (16:0/18:2) levels	35	−0.06	0.018	0.946	0.913	0.980	0.002
Succinate levels	26	0.04	0.014	1.040	1.012	1.069	0.005
1‐Methylnicotinamide levels	19	0.07	0.023	1.068	1.021	1.117	0.004
X‐12026 levels	27	−0.06	0.020	0.943	0.906	0.981	0.003

Abbreviations: 3‐CMPFP, 3‐carboxy‐4‐methyl‐5‐pentyl‐2‐furanpropionate; 5AAA, 5alpha‐androstan‐3alpha; B, beta‐value; IVW, inverse‐variance weighted; LCI, lower confidence interval; Nsnp, number of SNPs; OR, odds ratio; Se, standard error; Snp, single nucleotide polymorphism; UCI, upper confidence interval.

### 3.5 Impact of ICTs on PMs

According to the 13 types of ICTs and 27 types of PMs screened in the previous article, we analyzed the causal relationship between the two. MR analysis revealed 11 causal relationships, including CD25 on IgD+ CD24− and 1−stearoyl−2−linoleoyl−GPE (18:0/18:2) levels (OR = 1.021 [1.001 to 1.042], *p* = 0.042), CD3 on CD28+ CD4+ and 1−myristoyl−2−arachidonoyl−GPC (14:0/20:4) levels (OR = 1.055 [1.017 to 1.093], *p* = 0.037), etc. No heterogeneity or horizontal pleiotropy was observed, indicating that specific SNPs do not drive the causal estimates.

### 3.6 Mediated Effect of ICTs on Asthma

**FIGURE 6:**
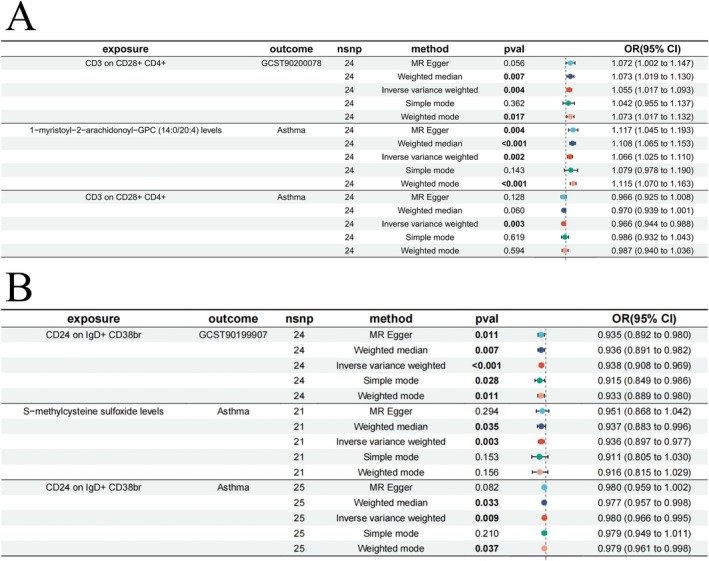
Forest plot of mediated effect.

We used the causal relationship of the 11 ICTs and PMs obtained above to calculate the mediated effects to determine their influence on the outcome factors. The mediation analysis revealed that there are two pathways involved in the mediation process (Figure 6, Table 3): −9.81% (−1.2%, −18.4%) of the effect of CD24 on IgD+ CD38br on asthma is mediated by S‐methylcysteine sulfoxide levels, with the mediated effect value (*p* = 0.006) being 0.003 (0.0004, 0.006); −21.4%(−6.2%, −36.6%) of the effect of CD3 on CD28+ CD4+ on asthma is mediated by 1‐myristoyl‐2‐arachidonoyl‐GPC (14:0/20:4) levels, with the mediated effect value (*p* = 0.025) being 0.004 (0.001, 0.007). Figure 6 illustrates the mechanisms of the two mediation pathways.

**TABLE 3:** Mediation Mendelian randomization analysis of the causal relationship between immune cell traits, plasma metabolites, and asthma.

Immune cells	Plasma metabolites	ME	MP	*p*‐value
CD24 on IgD+ CD38br	S−methylcysteine sulfoxide levels	0.004	−21.4%	0.006
CD3 on CD28+ CD4+	1‐Myristoyl‐2‐arachidonoyl‐GPC (14:0/20:4) levels	0.003	−9.81%	0.026

Abbreviations: ME, mediated effect; MP, mediated proportion.

## 4 Discussion

As a persistent illness with a considerable impact on worldwide health, asthma presents various treatment difficulties. Statistics show that over 300 million people around the world have asthma, with projections suggesting that the number of cases will increase by 2030. Managing asthma remains difficult due to the disease's heterogeneous nature and the variability in how patients respond to treatments. Current therapies often focus on symptom control without addressing underlying causal factors, highlighting the need for novel therapeutic targets. Leveraging genetic variants as IVs, MR offers a robust approach to uncovering causality between biomarkers and disease outcomes. By leveraging this methodological advantage, we can effectively counteract the confounding and reverse causation biases typical of observational studies [20]. Several studies akin to ours have utilized MR to investigate mediation effects of biomarkers on asthma. For instance, Chen et al. explored the mediation pathways of gut microbiota and immune cell in non‐small cell lung cancer severity using MR [21]. Similarly, Yan et al. conducted MR analysis to elucidate the role of gut microbiome and PMs in asthma pathogenesis [22]. We have found several clinical studies investigating the role of PMs in asthma, and the findings from these studies show certain similarities to our own results. These studies have highlighted the potential involvement of specific PMs in the onset and progression of asthma, particularly in relation to immune modulation, airway inflammation, and other key pathophysiological processes. The similarity of these results to our findings not only helps to contextualize our work within the broader landscape of asthma research but also strengthens the biological plausibility of our conclusions. By integrating these findings, we are able to gain a deeper understanding of the complex metabolic pathways underlying asthma, thus supporting the hypothesis that PMs could serve as crucial biomarkers for disease progression or potential therapeutic targets. This alignment between studies further underscores the relevance and potential impact of our research in advancing the management of asthma [23, 24, 25].

In the context of asthma, immune cells are crucial in regulating airway inflammation and hyperreactivity. Dysregulation of these immune responses leads to persistent inflammation, mucus production, and changes in airway structure, which are typical of the disease. Our study identified several ICTs with significant causal links to asthma. Significant markers associated with a higher asthma risk include CD14 on CD33br HLA DR+ CD14dim, commonly found on MDSCs and macrophages. MDSCs are known to have immunosuppressive effects and promote inflammation by producing cytokines and modulating T cell responses [26]. Monocytes and macrophages are pivotal in exacerbating airway inflammation and tissue remodeling. By generating pro‐inflammatory cytokines, they not only drive the inflammatory process but also inflict damage on the airway tissues, contributing to chronic and progressive airway disorders [27]. Among the immune cell markers linked to a lower risk of asthma are several subsets that play crucial roles in immune modulation and tolerance. CD24 expression on IgD+ CD38br B cells, for instance, characterizes a regulatory B cell subset capable of secreting anti‐inflammatory cytokines and promoting immune tolerance [28]. Recent literature supports these findings, highlighting the dynamic interplay between immune cell subsets and asthma severity. The results emphasize the potential for immune modulation therapies to correct imbalances in the immune system of individuals with asthma.

PMs are vital in regulating the metabolic and inflammatory pathways that contribute to the development of asthma. Altered metabolic profiles can significantly influence oxidative stress levels, immune cell function, and airway inflammation, thereby impacting disease progression and treatment outcomes. Our MR analysis revealed 27 PMs with causality to asthma. PMs such as succinate, androsterone sulfate, and 1‐myristoyl‐2‐arachidonoyl‐GPC (14:0/20:4) were associated with increased asthma risk, suggesting their involvement in exacerbating airway inflammation and hyperresponsiveness [29]. Conversely, protective PMs including 1,2‐dilinoleoyl‐GPC (18:2/18:2) and S‐methylcysteine sulfoxide may mitigate asthma severity by modulating inflammatory pathways [299]. These findings align with current research emphasizing the intricate interplay between metabolic dysregulation and asthma pathogenesis. Focusing on these metabolic pathways offers potential for creating new therapeutic strategies to improve asthma management and patient outcomes.

Mediation analysis provides valuable insights into how ICTs and PMs mediate their effects on asthma outcomes through intermediate pathways. By quantifying these mediating effects, we can elucidate complex interactions between biological markers and disease progression, offering avenues for targeted therapeutic interventions.

In our study, we observed significant mediation effects where 1‐myristoyl‐2‐arachidonoyl‐GPC (14:0/20:4) levels mediated a substantial proportion of the effect of CD3 on CD28+ CD4+ T cells on asthma risk. These findings underscore the intricate interplay between immune dysregulation and metabolic pathways in asthma pathophysiology, highlighting potential targets for therapeutic intervention aimed at disrupting disease mechanisms.

Both KEGG and SMPDB enrichment analysis revealed significant enrichment of PMs in glycine and serine. Glycine supports anti‐inflammatory responses and is a precursor to glutathione, a potent antioxidant. Elevated glycine levels may reduce oxidative stress and inflammation in asthma by enhancing glutathione synthesis and neutralizing reactive oxygen species [30]. Serine plays a role in sphingolipid biosynthesis and immune function. Alterations in serine metabolism can impact sphingolipid levels, affecting cell membrane integrity and inflammatory signaling. Furthermore, by influencing folate metabolism, serine may alter immune cell function and subsequently modulate inflammation in asthma [30]. In summary, the metabolism of glycine and serine affects inflammatory responses and oxidative stress in asthma, suggesting potential targets for personalized treatment strategies.

As far as we know, our research is the first to use MR to explore the causality among ICTs, PMs, and asthma, along with the mediating effects of PMs. Additionally, MR analysis has its own advantage, which is controlling confounding factors. Nevertheless, one notable limitation of this study is that all genome‐wide association summary statistics used for the exposure and outcome datasets were derived exclusively from individuals of European ancestry. As a result, the generalizability of our findings to non‐European populations may be limited, given known differences in genetic architecture, environmental exposures, and asthma prevalence across ethnic groups. Future studies are needed to replicate these findings in multiethnic or non‐European cohorts to confirm whether the identified causal associations hold consistently across diverse populations.

## 5 Conclusion

Thirteen ICTs were found to have a causal relationship with asthma, and there are 27 PMs that can influence asthma. We found that ICTs (CD24 on IgD+ CD38br and CD3 on CD28+ CD4+) influence asthma through two pathways, mediated by PMs (S‐methylcysteine sulfoxide levels and 1‐myristoyl‐2‐arachidonoyl‐GPC (14:0/20:4) levels). These outcomes have the potential to identify key therapeutic targets for creating customized asthma treatment plans.

## Author Contributions

Wenxiong Zhang had full access to all the data in the manuscript and takes responsibility for the integrity of the data and the accuracy of the data analysis. All authors read and approved the final manuscript.

Concept and design: Zhuozheng Hu, Peihao Xu, Jiajun Wu, Weijun Zhou, Yajie Zhou, Lei Xie, Yong Cheng, and Wenxiong Zhang. Acquisition, analysis, or interpretation of data: Zhuozheng Hu, Peihao Xu, Jiajun Wu, Weijun Zhou, Yajie Zhou, Lei Xie, Yong Cheng, and Wenxiong Zhang. Statistical analysis: Zhuozheng Hu, Jiajun Wu, Yong Cheng, and Wenxiong Zhang. Drafting of the manuscript: Zhuozheng Hu, Jiajun Wu, Peihao Xu, Weijun Zhou, Yajie Zhou, Lei Xie, Yong Cheng, and Wenxiong Zhang. Critical revision of the manuscript for important intellectual content: Zhuozheng Hu, Yong Cheng, and Wenxiong Zhang. Supervision: Zhuozheng Hu and Wenxiong Zhang.

## Ethics Statement

The authors have nothing to report.

## Consent

The authors have nothing to report.

## Conflicts of Interest

The authors declare no conflicts of interest.

## Supporting information


**Figure S1.** Scatter plots for the causal association between immune cell traits and asthma.

**Figure S2.** Leave‐one‐out plots for the causal association between immune cell traits and asthma.

**Figure S3.** Scatter plots for the causal association between plasma metabolites and asthma with an increased risk.

**Figure S4.** Scatter plots for the causal association between plasma metabolites and asthma with a low risk.

**Figure S5.** Leave‐one‐out plots for the causal association between plasma metabolites and asthma with an increased risk.

**Figure S6.** Leave‐one‐out plots for the causal association between plasma metabolites and asthma with a low risk.

**Figure S7.** Enrichment analysis results of the causal plasma metabolites of asthma based on the SMPD (A‐B).

**Table S1.** Bonferroni correction of immune cell traits.

**Table S2.** The heterogeneity analysis of causality between immune cell traits and asthma based on IVW and MR Egger methods.

**Table S3.** The pleiotropy analysis of causality between immune cell traits and asthma based on MR results.

**Table S4.** Reverse MR analysis of asthma on immune cell traits.

**Table S5.** Bonferroni correction of plasma metabolites.

**Table S6.** The heterogeneity analysis of causality between plasma metabolites and asthma based on IVW and MR Egger methods.

**Table S7.** The pleiotropy analysis of causality between plasma metabolites and asthma based on MR results.

## Data Availability

The data for 731 ICTs (accession numbers for European GWASs: Ebi‐a‐GCST0001391 to Ebi‐a‐GCST0002121) were sourced from the GWAS catalog. Data on PMs was from a cohort of 7824 individuals of European descent sourced from the GWAS database (accession numbers for European GWASs: GCST90199621‐90201020). Data on asthma as an outcome factor were selected from the GWAS database (accession numbers for European GWASs: GCST90018795). All data in our study is publicly available.
