# Supplementary figures and images for: Causal Relationships Between Immune Cell Traits, Plasma Metabolites, and Asthma: A Two‐Step, Two‐Sample Mendelian Randomization Study
Source: Clin Respir J. 2025 Jun 23;19(6):e70097. doi: 10.1111/crj.70097 (PMC12185225; doi:10.1111/crj.70097)

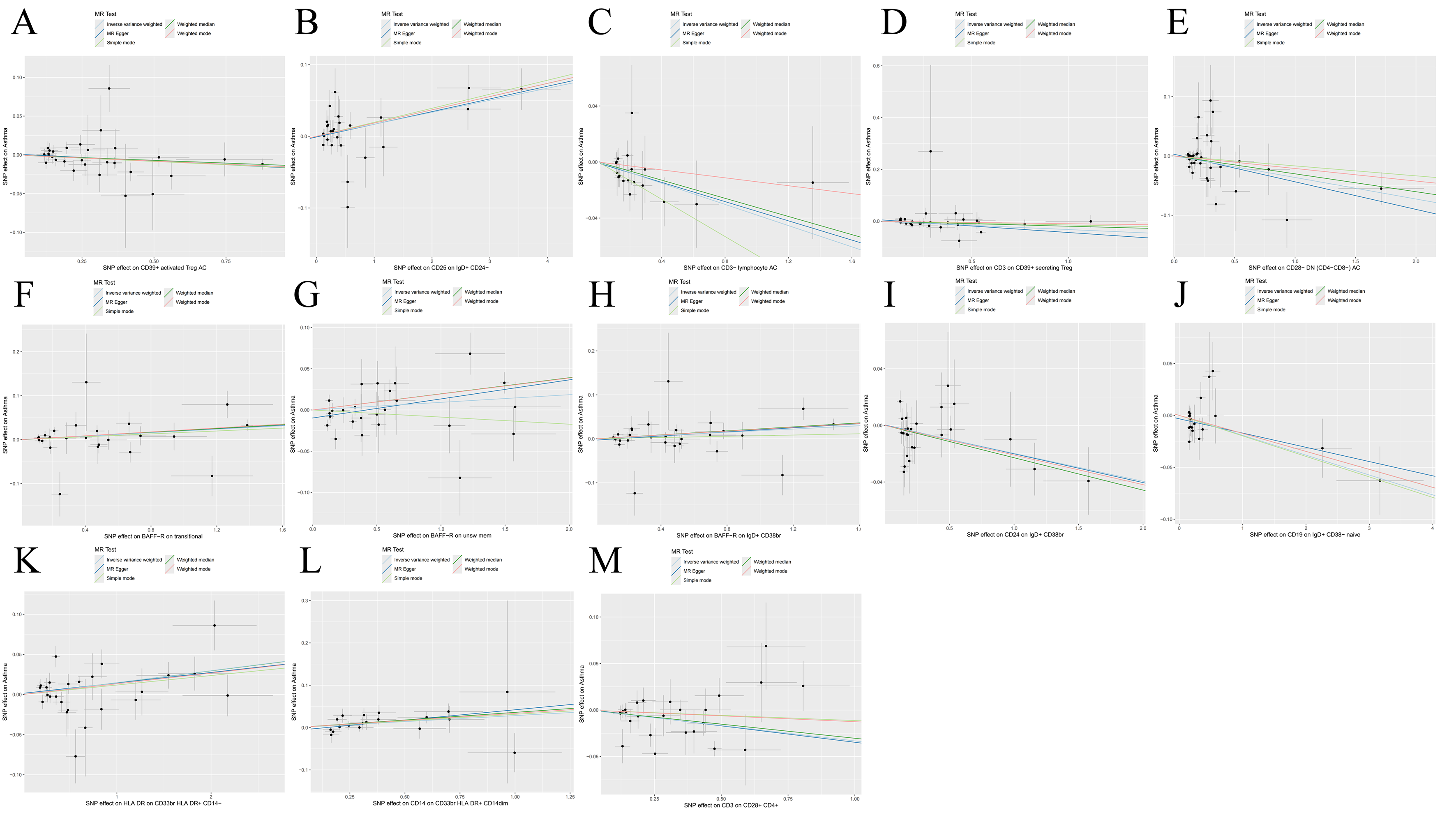

Supplement: Supplementary file 1 — Figure S1. Scatter plots for the causal association between immune cell traits and asthma. [file CRJ-19-e70097-s007.tif]

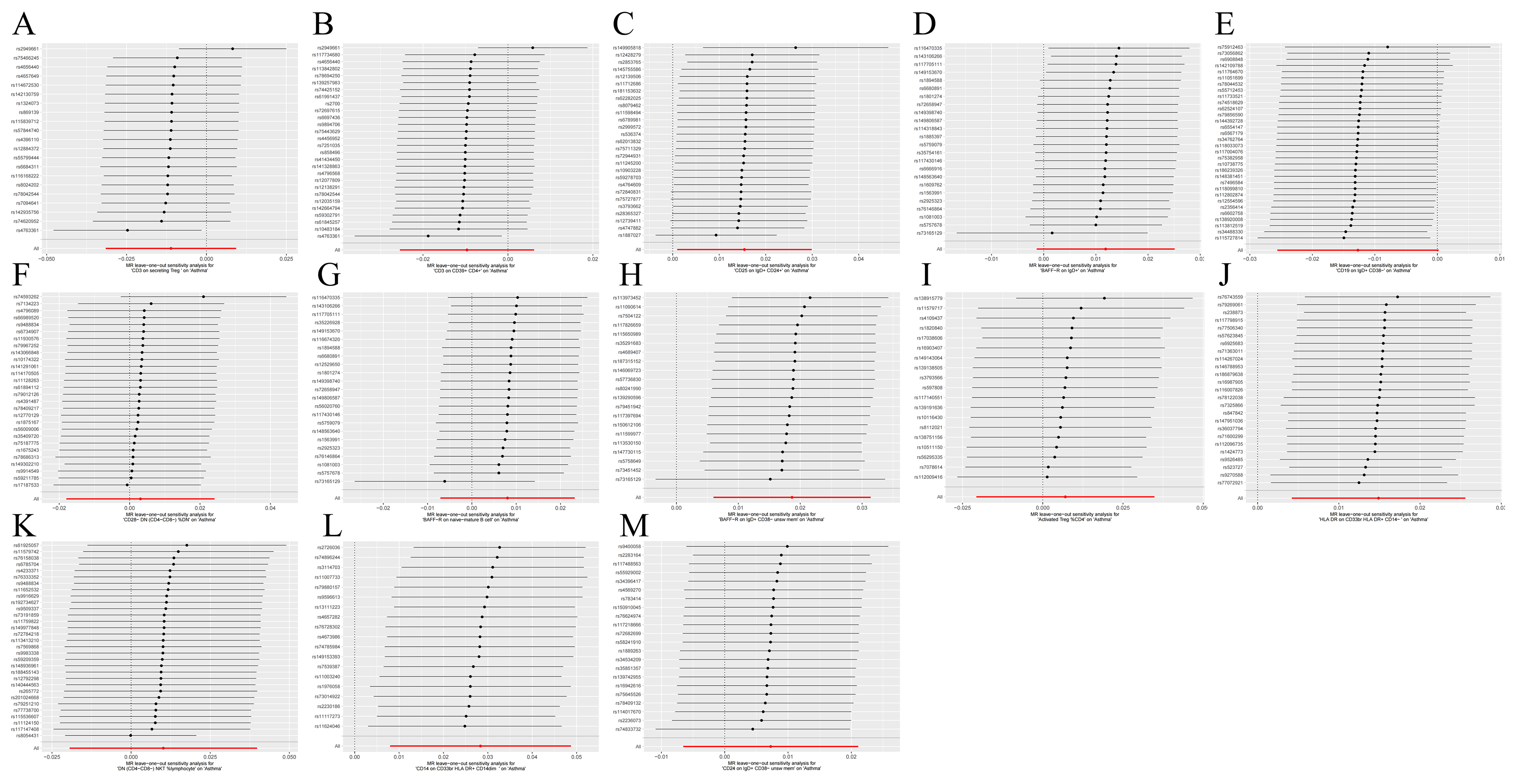

Supplement: Supplementary file 2 — Figure S2. Leave‐one‐out plots for the causal association between immune cell traits and asthma. [file CRJ-19-e70097-s013.tif]

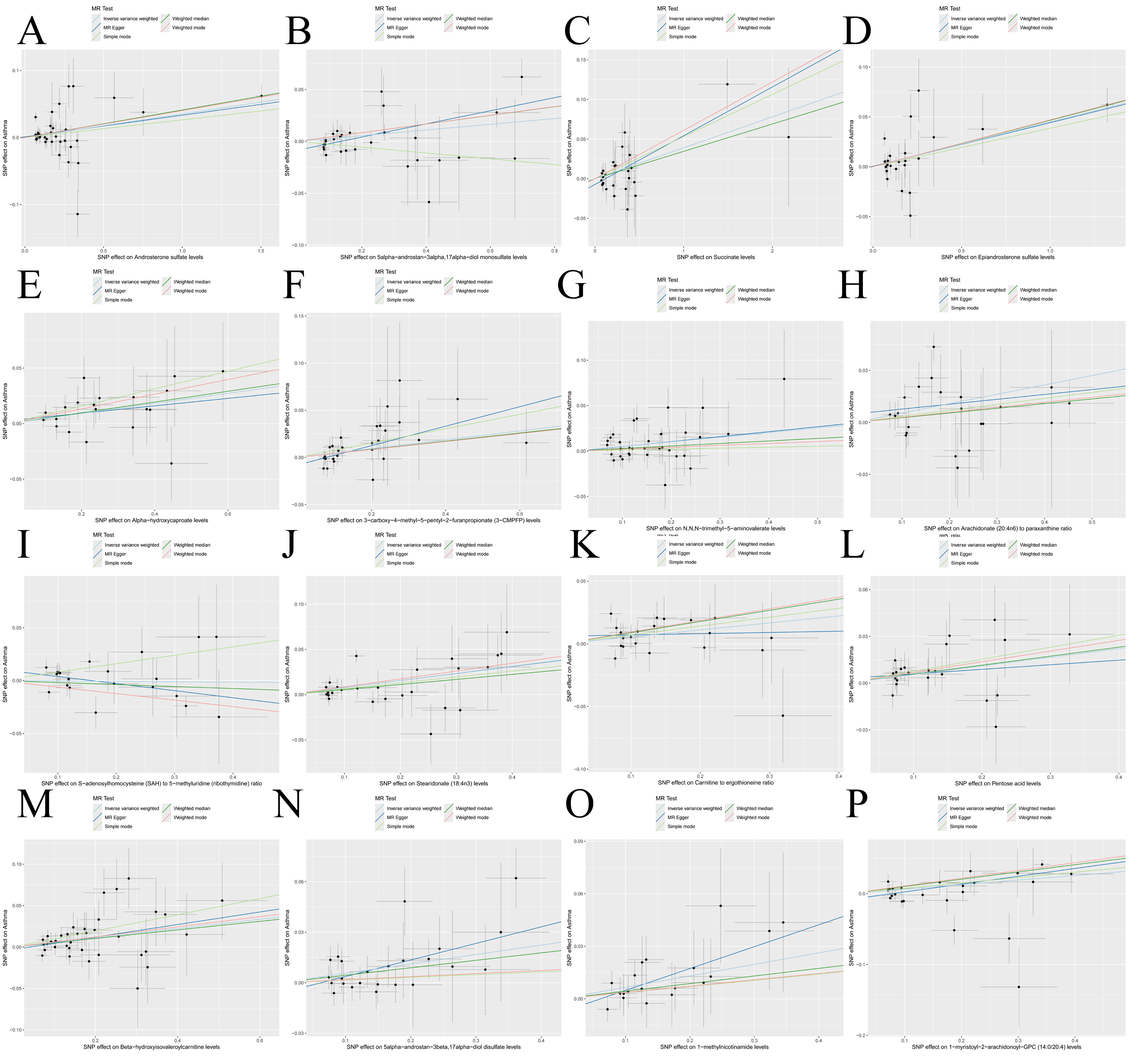

Supplement: Supplementary file 3 — Figure S3. Scatter plots for the causal association between plasma metabolites and asthma with an increased risk. [file CRJ-19-e70097-s002.tif]

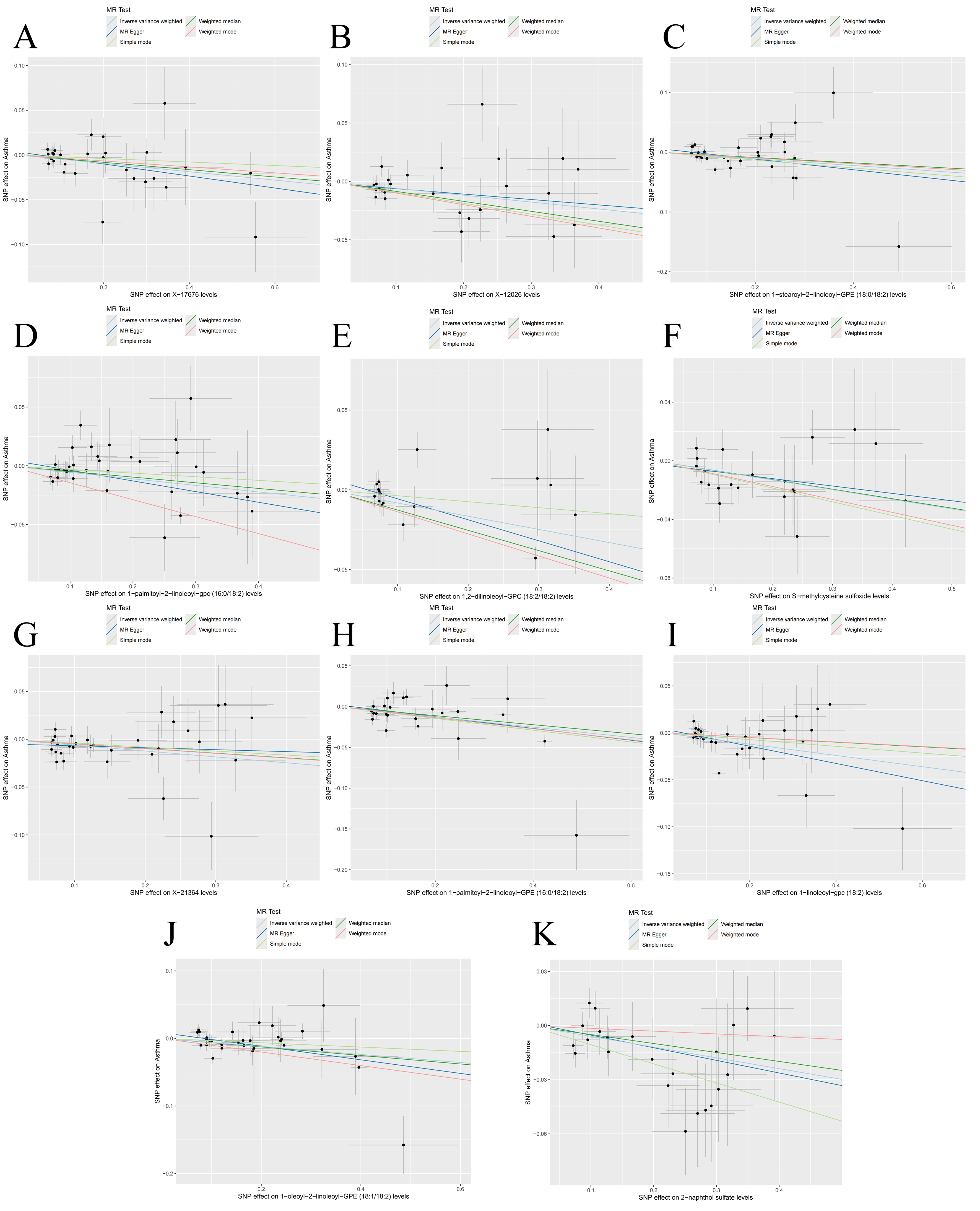

Supplement: Supplementary file 4 — Figure S4. Scatter plots for the causal association between plasma metabolites and asthma with a low risk. [file CRJ-19-e70097-s011.tif]

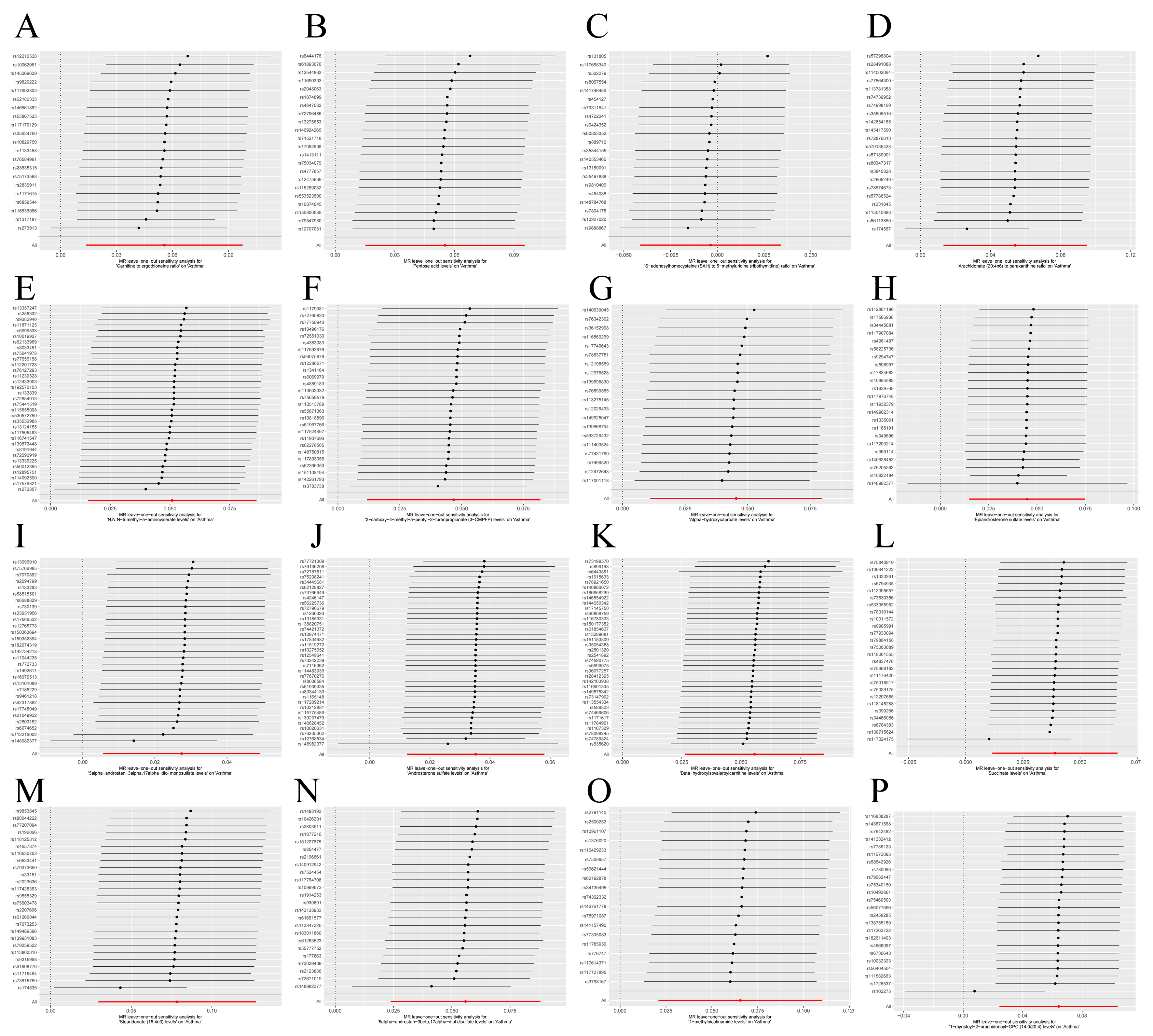

Supplement: Supplementary file 5 — Figure S5. Leave‐one‐out plots for the causal association between plasma metabolites and asthma with an increased risk. [file CRJ-19-e70097-s008.tif]

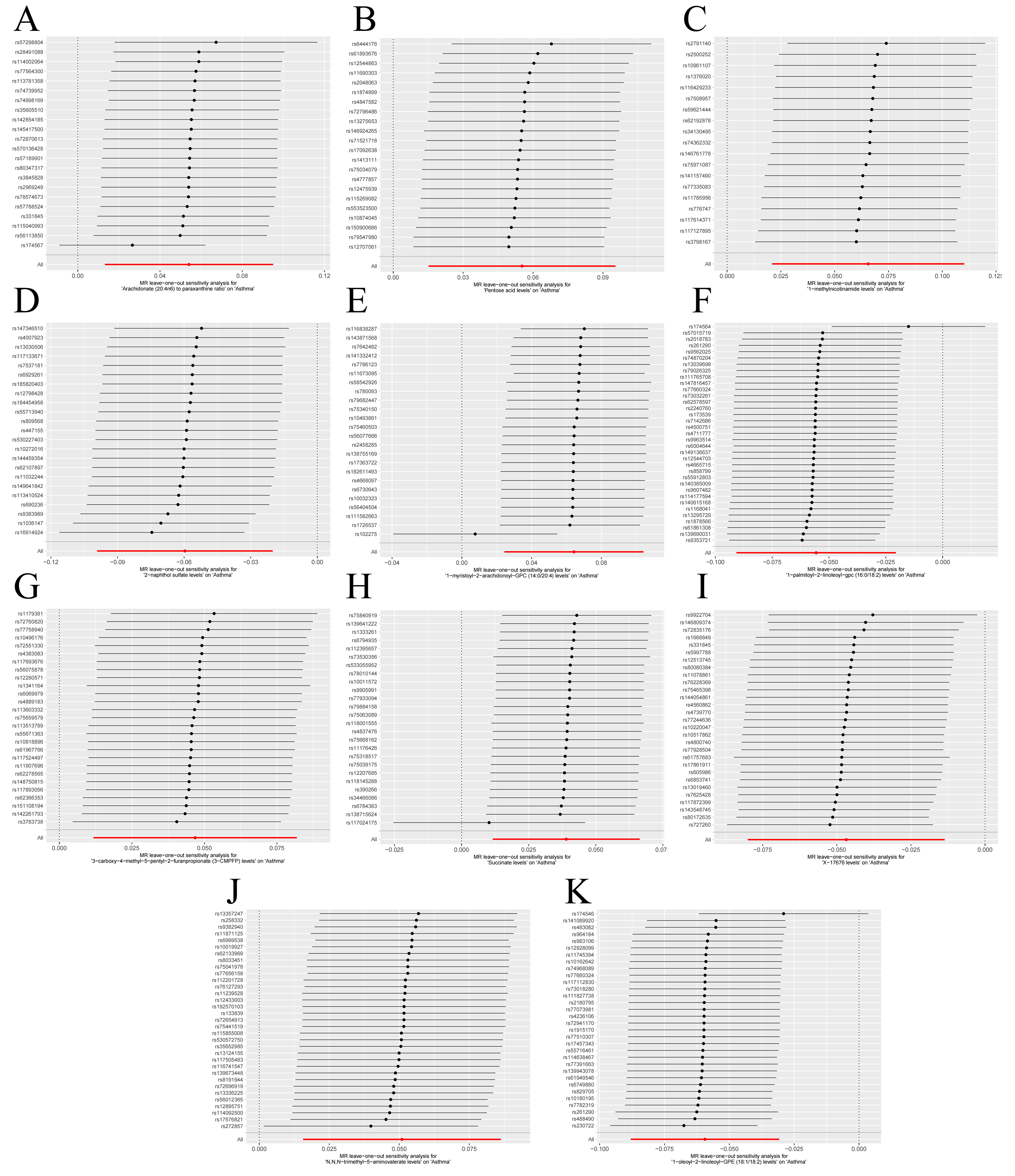

Supplement: Supplementary file 6 — Figure S6. Leave‐one‐out plots for the causal association between plasma metabolites and asthma with a low risk. [file CRJ-19-e70097-s005.tif]

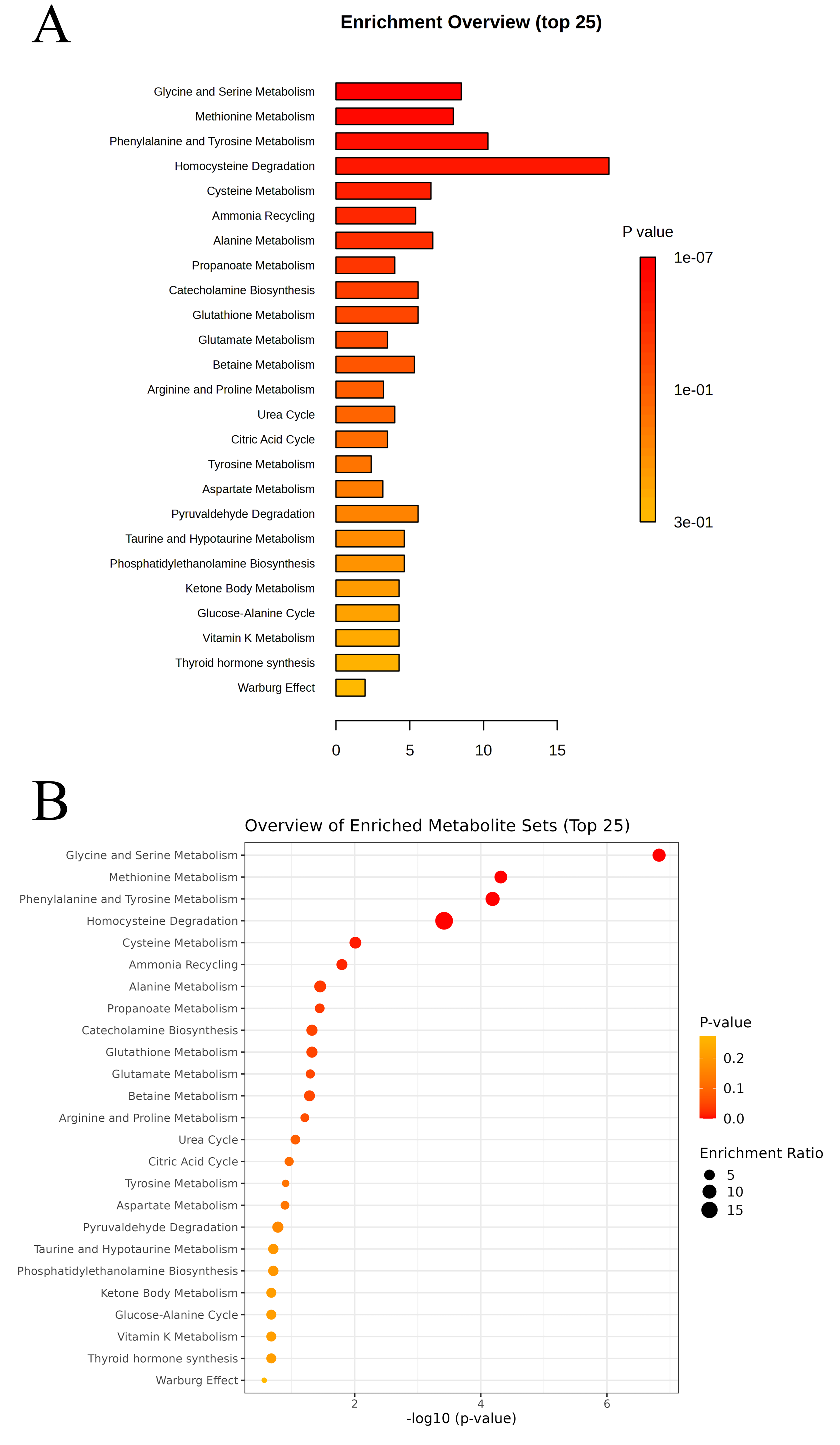

Supplement: Supplementary file 7 — Figure S7. Enrichment analysis results of the causal plasma metabolites of asthma based on the SMPD (A‐B). [file CRJ-19-e70097-s010.tif]
