# Supplementary material for: Causal Relationships Between Immune Cell Traits, Plasma Metabolites, and Asthma: A Two‐Step, Two‐Sample Mendelian Randomization Study
Source: Clin Respir J. 2025 Jun 23;19(6):e70097. doi: 10.1111/crj.70097 (PMC12185225; doi:10.1111/crj.70097)
Supplement: Supplementary file 8 — Table S1. Bonferroni correction of immune cell traits. [file CRJ-19-e70097-s001.docx]

**Table S1** Bonferroni correction of immune cell traits.

| **Exposure** | **Bonferroni-corrected p-value** | **Bonferroni Significant?** |
| --- | --- | --- |
| CD39+ activated Treg AC | 0.006 | No (Nominally significant) |
| CD3- lymphocyte AC | 0.005 | No (Nominally significant) |
| CD28- DN (CD4-CD8-) AC | 0.008 | No (Nominally significant) |
| BAFF-R on IgD+ CD38- unsw mem | 0.004 | No (Nominally significant) |
| BAFF-R on IgD+ CD38br | 0.006 | No (Nominally significant) |
| BAFF-R on transitional | 0.008 | No (Nominally significant) |
| CD19 on IgD+ CD38- naive | 0.008 | No (Nominally significant) |
| CD24 on IgD+ CD38br | 0.009 | No (Nominally significant) |
| CD25 on IgD+ CD24- | 0.002 | Yes (Bonferroni significant) |
| CD3 on CD39+ secreting Treg | 0.000326 | Yes (Bonferroni significant) |
| CD3 on CD28+ CD4+ | 0.004 | No (Nominally significant) |
| CD14 on CD33br HLA DR+ CD14dim | 0.006 | No (Nominally significant) |
| HLA DR on CD33br HLA DR+ CD14- | 0.006 | No (Nominally significant) |

**Bonferroni-corrected significance threshold**: P < 0.0038 (0.05/13).
