# Supplementary material for: Causal Relationships Between Immune Cell Traits, Plasma Metabolites, and Asthma: A Two‐Step, Two‐Sample Mendelian Randomization Study
Source: Clin Respir J. 2025 Jun 23;19(6):e70097. doi: 10.1111/crj.70097 (PMC12185225; doi:10.1111/crj.70097)
Supplement: Supplementary file 9 — Table S2. The heterogeneity analysis of causality between immune cell traits and asthma based on IVW and MR Egger methods. [file CRJ-19-e70097-s014.docx]

**Table S2** The heterogeneity analysis of causality between immune cell traits and asthma based on IVW and MR Egger methods.

| **Exposure** | **Method** | **Q** | **Q_df** | **Q_pval** |
| --- | --- | --- | --- | --- |
| CD39+ activated Treg AC | MR Egger | 27.519 | 28 | 0.490 |
| CD39+ activated Treg AC | IVW | 27.737 | 29 | 0.532 |
| CD3- lymphocyte AC | MR Egger | 9.521 | 16 | 0.890 |
| CD3- lymphocyte AC | IVW | 9.548 | 17 | 0.921 |
| CD28- DN (CD4-CD8-) AC | MR Egger | 67.156 | 30 | 1.54E-04 |
| CD28- DN (CD4-CD8-) AC | IVW | 67.891 | 31 | 1.43E-04 |
| BAFF-R on IgD+ CD38- unsw mem | MR Egger | 20.944 | 19 | 0.340 |
| BAFF-R on IgD+ CD38- unsw mem | IVW | 21.920 | 20 | 0.345 |
| BAFF-R on IgD+ CD38br | MR Egger | 33.405 | 25 | 0.121 |
| BAFF-R on IgD+ CD38br | IVW | 34.129 | 26 | 0.131 |
| BAFF-R on transitional | MR Egger | 31.248 | 22 | 0.091 |
| BAFF-R on transitional | IVW | 31.261 | 23 | 0.117 |
| CD19 on IgD+ CD38- naive | MR Egger | 20.305 | 17 | 0.259 |
| CD19 on IgD+ CD38- naive | IVW | 21.426 | 18 | 0.258 |
| CD24 on IgD+ CD38br | MR Egger | 24.172 | 23 | 0.394 |
| CD24 on IgD+ CD38br | IVW | 24.177 | 24 | 0.452 |
| CD25 on IgD+ CD24- | MR Egger | 27.890 | 24 | 0.265 |
| CD25 on IgD+ CD24- | IVW | 27.977 | 25 | 0.309 |
| CD3 on CD39+ secreting Treg | MR Egger | 32.694 | 26 | 0.171 |
| CD3 on CD39+ secreting Treg | IVW | 35.365 | 27 | 0.130 |
| CD3 on CD28+ CD4+ | MR Egger | 36.830 | 23 | 0.034 |
| CD3 on CD28+ CD4+ | IVW | 36.850 | 24 | 0.045 |
| CD14 on CD33br HLA DR+ CD14dim | MR Egger | 20.899 | 17 | 0.231 |
| CD14 on CD33br HLA DR+ CD14dim | IVW | 22.206 | 18 | 0.223 |
| CD11b on Gr MDSC | MR Egger | 0.501 | 1 | 0.479 |
| CD11b on Gr MDSC | IVW | 0.687 | 2 | 0.709 |
| HLA DR on CD33br HLA DR+ CD14- | MR Egger | 37.942 | 23 | 0.026 |
| HLA DR on CD33br HLA DR+ CD14- | IVW | 38.079 | 24 | 0.034 |

**Abbreviations:** IVW: Inverse-variance weighted; MR: Mendelian randomization.
