# Supplementary material for: Causal Relationships Between Immune Cell Traits, Plasma Metabolites, and Asthma: A Two‐Step, Two‐Sample Mendelian Randomization Study
Source: Clin Respir J. 2025 Jun 23;19(6):e70097. doi: 10.1111/crj.70097 (PMC12185225; doi:10.1111/crj.70097)
Supplement: Supplementary file 10 — Table S3. The pleiotropy analysis of causality between immune cell traits and asthma based on MR results. [file CRJ-19-e70097-s009.docx]

**Table S3** The pleiotropy analysis of causality between immune cell traits and asthma based on MR results.

| **Exposure** | **Egger intercept** | **Se** | **P-value** |
| --- | --- | --- | --- |
| CD39+ activated Treg AC | 0.001 | 0.003 | 0.644 |
| CD3- lymphocyte AC | -7.98E-04 | 0.005 | 0.871 |
| CD28- DN (CD4-CD8-) AC | 0.003 | 0.005 | 0.571 |
| BAFF-R on IgD+ CD38- unsw mem | -0.005 | 0.005 | 0.358 |
| BAFF-R on IgD+ CD38br | -0.003 | 0.004 | 0.468 |
| BAFF-R on transitional | -4.10E-04 | 0.004 | 0.925 |
| CD19 on IgD+ CD38- naive | -0.003 | 0.003 | 0.346 |
| CD24 on IgD+ CD38br | 2.44E-04 | 0.003 | 0.944 |
| CD25 on IgD+ CD24- | -9.88E-04 | 0.004 | 0.786 |
| CD3 on CD39+ secreting Treg | 0.006 | 0.004 | 0.157 |
| CD3 on CD28+ CD4+ | 6.62E-04 | 0.006 | 0.911 |
| CD14 on CD33br HLA DR+ CD14dim | -0.007 | 0.007 | 0.317 |
| CD11b on Gr MDSC | 0.025 | 0.0597 | 0.740 |
| HLA DR on CD33br HLA DR+ CD14- | 0.001 | 0.005 | 0.776 |

**Abbreviations:** MR: Mendelian randomization; Se: Standard error.
