# Supplementary material for: Causal Relationships Between Immune Cell Traits, Plasma Metabolites, and Asthma: A Two‐Step, Two‐Sample Mendelian Randomization Study
Source: Clin Respir J. 2025 Jun 23;19(6):e70097. doi: 10.1111/crj.70097 (PMC12185225; doi:10.1111/crj.70097)
Supplement: Supplementary file 11 — Table S4. Reverse MR analysis of asthma on immune cell traits. [file CRJ-19-e70097-s003.docx]

**Table S4** Reverse Mendelian randomization analysis of lung adenocarcinoma on immune cells.

| **Exposure** | **Method** | **Nsnp** | **B** | **Se** | **OR** | **Rev P-value** |
| --- | --- | --- | --- | --- | --- | --- |
| Plasmacytoid DC %DC | IVW | 21 | 0.10 | 0.03 | 1.10 | 0.73 |
| CD39+ secreting Treg %secreting Treg | IVW | 20 | -0.07 | 0.03 | 0.93 | 0.61 |
| Naive CD8br %CD8br | IVW | 24 | -0.09 | 0.03 | 0.91 | 0.65 |
| T cell %leukocyte | IVW | 15 | -0.10 | 0.03 | 0.91 | 0.56 |
| Granulocyte %leukocyte | IVW | 21 | 0.13 | 0.05 | 1.14 | 0.39 |
| CD28+ CD45RA+ CD8dim %T cell | IVW | 33 | -0.04 | 0.01 | 0.96 | 0.81 |
| CD28+ CD45RA+ CD8dim AC | IVW | 35 | -0.03 | 0.01 | 0.97 | 0.59 |
| CD28- CD127- CD25++ CD8br %T cell | IVW | 14 | -0.17 | 0.06 | 0.84 | 0.20 |
| CD19 on IgD+ CD38br | IVW | 13 | -0.18 | 0.05 | 0.83 | 0.42 |
| CD19 on IgD- CD27- | IVW | 22 | -0.16 | 0.04 | 0.85 | 0.76 |
| CD25 on CD45RA+ CD4 not Treg | IVW | 20 | 0.12 | 0.04 | 1.12 | 0.86 |
| CD25 on resting Treg | IVW | 14 | -0.12 | 0.04 | 0.89 | 0.83 |
| CD25 on CD4+ | IVW | 12 | -0.13 | 0.04 | 0.88 | 0.58 |
| CCR2 on granulocyte | IVW | 16 | 0.15 | 0.04 | 1.16 | 0.48 |

**Abbreviations:** B: Beta-value; IVW: Inverse-variance weighted; LUAD: Lung adenocarcinoma; Nsnp: Number of SNPs; OR: Odds ratio; Snp: Single nucleotide polymorphism; Se: Standard error.
