# Supplementary material for: Causal Relationships Between Immune Cell Traits, Plasma Metabolites, and Asthma: A Two‐Step, Two‐Sample Mendelian Randomization Study
Source: Clin Respir J. 2025 Jun 23;19(6):e70097. doi: 10.1111/crj.70097 (PMC12185225; doi:10.1111/crj.70097)
Supplement: Supplementary file 12 — Table S5. Bonferroni correction of plasma metabolites. [file CRJ-19-e70097-s006.docx]

**Table S5** Bonferroni correction of plasma metabolites.

| **Exposure** | **Bonferroni-corrected p-value** | **Bonferroni Significant?** |
| --- | --- | --- |
| Stearidonate (18:4n3) levels | 0.054 | No (Nominally significant) |
| 1-linoleoyl-gpc (18:2) levels | 0.189 | No (Nominally significant) |
| Epiandrosterone sulfate levels | 0.081 | No (Nominally significant) |
| Beta-hydroxyisovaleroylcarnitine levels | 0.00537 | Yes (Bonferroni significant) |
| Alpha-hydroxycaproate levels | 0.270 | No (Nominally significant) |
| 1-palmitoyl-2-linoleoyl-GPE (16:0/18:2) levels | 0.00063 | Yes (Bonferroni significant) |
| 5alpha-androstan-3beta,17alpha-diol disulfate levels | 0.0173 | No (Nominally significant) |
| 5alpha-androstan-3alpha,17beta-diol monosulfate (1) levels | 0.108 | No (Nominally significant) |
| S-methylcysteine sulfoxide levels | 0.081 | No (Nominally significant) |
| 1,2-dilinoleoyl-GPC (18:2/18:2) levels | 0.01053 | No (Nominally significant) |
| 1-stearoyl-2-linoleoyl-GPE (18:0/18:2) levels | 0.108 | No (Nominally significant) |
| 1-myristoyl-2-arachidonoyl-GPC (14:0/20:4) levels | 0.054 | No (Nominally significant) |
| 1-oleoyl-2-linoleoyl-GPE (18:1/18:2) levels | 0.00126 | Yes (Bonferroni significant) |
| N,N,N-trimethyl-5-aminovalerate levels | 0.135 | No (Nominally significant) |
| 3-carboxy-4-methyl-5-pentyl-2-furanpropionate (3-CMPFP) levels | 0.243 | No (Nominally significant) |
| 2-naphthol sulfate levels | 0.081 | No (Nominally significant) |
| Pentose acid levels | 0.189 | No (Nominally significant) |
| 1-palmitoyl-2-linoleoyl-gpc (16:0/18:2) levels | 0.054 | No (Nominally significant) |
| Succinate levels | 0.135 | No (Nominally significant) |
| 1-methylnicotinamide levels | 0.108 | No (Nominally significant) |
| X-12026 levels | 0.081 | No (Nominally significant) |
| X-17676 levels | 0.162 | No (Nominally significant) |
| X-21364 levels | 0.081 | No (Nominally significant) |
| Androsterone sulfate levels | 0.081 | No (Nominally significant) |
| S-adenosylhomocysteine (SAH) to leucine ratio | 0.054 | No (Nominally significant) |
| Carnitine to ergothioneine ratio | 0.243 | No (Nominally significant) |
| Arachidonate (20:4n6) to paraxanthine ratio | 0.270 | No (Nominally significant) |

**Bonferroni-corrected significance threshold**: P < 0.00185 (0.05/27).
