# Supplementary material for: Causal Relationships Between Immune Cell Traits, Plasma Metabolites, and Asthma: A Two‐Step, Two‐Sample Mendelian Randomization Study
Source: Clin Respir J. 2025 Jun 23;19(6):e70097. doi: 10.1111/crj.70097 (PMC12185225; doi:10.1111/crj.70097)
Supplement: Supplementary file 13 — Table S6. The heterogeneity analysis of causality between plasma metabolites and asthma based on IVW and MR Egger methods. [file CRJ-19-e70097-s004.docx]

**Table S6** The heterogeneity analysis of causality between plasma metabolites and asthma based on IVW and MR Egger methods.

| **Exposure** | **Method** | **Q** | **Q_df** | **Q_pval** |
| --- | --- | --- | --- | --- |
| Stearidonate (18:4n3) levels | MR Egger | 39.716 | 24 | 0.023 |
| Stearidonate (18:4n3) levels | IVW | 39.717 | 25 | 0.031 |
| 1-linoleoyl-gpc (18:2) levels | MR Egger | 45.418 | 28 | 0.020 |
| 1-linoleoyl-gpc (18:2) levels | IVW | 46.376 | 29 | 0.022 |
| Epiandrosterone sulfate levels | MR Egger | 40.339 | 21 | 0.007 |
| Epiandrosterone sulfate levels | IVW | 40.359 | 22 | 0.010 |
| Beta-hydroxyisovaleroylcarnitine levels | MR Egger | 31.376 | 34 | 0.597 |
| Beta-hydroxyisovaleroylcarnitine levels | IVW | 31.875 | 35 | 0.620 |
| Alpha-hydroxycaproate levels | MR Egger | 11.652 | 18 | 0.865 |
| Alpha-hydroxycaproate levels | IVW | 11.785 | 19 | 0.895 |
| 1-palmitoyl-2-linoleoyl-GPE (16:0/18:2) levels | MR Egger | 50.733 | 23 | 7.38E-04 |
| 1-palmitoyl-2-linoleoyl-GPE (16:0/18:2) levels | IVW | 51.146 | 24 | 0.001 |
| 5alpha-androstan-3beta,17alpha-diol disulfate levels | MR Egger | 20.218 | 22 | 0.569 |
| 5alpha-androstan-3beta,17alpha-diol disulfate levels | IVW | 21.691 | 23 | 0.539 |
| 5AAA,17beta-diol monosulfate (1) levels | MR Egger | 22.239 | 19 | 0.273 |
| 5AAA,17beta-diol monosulfate (1) levels | IVW | 22.898 | 20 | 0.294 |
| S-methylcysteine sulfoxide levels | MR Egger | 27.009 | 27 | 0.463 |
| S-methylcysteine sulfoxide levels | IVW | 27.223 | 28 | 0.506 |
| 1,2-dilinoleoyl-GPC (18:2/18:2) levels | MR Egger | 20.007 | 19 | 0.394 |
| 1,2-dilinoleoyl-GPC (18:2/18:2) levels | IVW | 20.156 | 20 | 0.448 |
| 1-stearoyl-2-linoleoyl-GPE (18:0/18:2) levels | MR Egger | 25.725 | 15 | 0.041 |
| 1-stearoyl-2-linoleoyl-GPE (18:0/18:2) levels | IVW | 29.514 | 16 | 0.021 |
| 1-myristoyl-2-arachidonoyl-GPC (14:0/20:4) levels | MR Egger | 72.928 | 28 | 7.18E-06 |
| 1-myristoyl-2-arachidonoyl-GPC (14:0/20:4) levels | IVW | 75.378 | 29 | 5.38E-06 |
| 1-oleoyl-2-linoleoyl-GPE (18:1/18:2) levels | MR Egger | 39.264 | 22 | 0.013 |
| 1-oleoyl-2-linoleoyl-GPE (18:1/18:2) levels | IVW | 44.176 | 23 | 0.005 |
| N, N, N-trimethyl-5-aminovalerate levels | MR Egger | 47.640 | 30 | 0.022 |
| N, N, N-trimethyl-5-aminovalerate levels | IVW | 53.497 | 31 | 0.007 |
| 3-CMPFP levels | MR Egger | 37.402 | 31 | 0.199 |
| 3-CMPFP levels | IVW | 37.410 | 32 | 0.235 |
| 2-naphthol sulfate levels | MR Egger | 18.048 | 25 | 0.840 |
| 2-naphthol sulfate levels | IVW | 20.983 | 26 | 0.743 |
| Pentose acid levels | MR Egger | 23.125 | 21 | 0.337 |
| Pentose acid levels | IVW | 23.220 | 22 | 0.389 |
| 1-palmitoyl-2-linoleoyl-gpc (16:0/18:2) levels | MR Egger | 15.753 | 20 | 0.732 |
| 1-palmitoyl-2-linoleoyl-gpc (16:0/18:2) levels | IVW | 16.061 | 21 | 0.766 |
| Succinate levels | MR Egger | 54.135 | 33 | 0.012 |
| Succinate levels | IVW | 56.011 | 34 | 0.010 |
| 1-methylnicotinamide levels | MR Egger | 16.214 | 24 | 0.880 |
| 1-methylnicotinamide levels | IVW | 20.110 | 25 | 0.741 |
| X-12026 levels | MR Egger | 7.407 | 17 | 0.978 |
| X-12026 levels | IVW | 8.487 | 18 | 0.970 |

**Abbreviations:** IVW: inverse-variance weighted; MR: Mendelian randomization; 3-CMPFP: 3-carboxy-4-methyl-5-pentyl-2-furanpropionate; 5AAA: 5alpha-androstan-3alpha.
