# Supplementary material for: Causal Relationships Between Immune Cell Traits, Plasma Metabolites, and Asthma: A Two‐Step, Two‐Sample Mendelian Randomization Study
Source: Clin Respir J. 2025 Jun 23;19(6):e70097. doi: 10.1111/crj.70097 (PMC12185225; doi:10.1111/crj.70097)
Supplement: Supplementary file 14 — Table S7. The pleiotropy analysis of causality between plasma metabolites and asthma based on MR results. [file CRJ-19-e70097-s012.docx]

**Table S7** The pleiotropy analysis of causality between plasma metabolites and asthma based on MR results.

| **Exposure** | **Egger intercept** | **Se** | **P-value** |
| --- | --- | --- | --- |
| Stearidonate (18:4n3) levels | 1.14E-04 | 0.006 | 0.985 |
| 1-linoleoyl-gpc (18:2) levels | 0.004 | 0.005 | 0.449 |
| Epiandrosterone sulfate levels | 4.14E-04 | 0.004 | 0.919 |
| Beta-hydroxyisovaleroylcarnitine levels | -0.003 | 0.004 | 0.485 |
| Alpha-hydroxycaproate levels | 0.003 | 0.007 | 0.720 |
| 1-palmitoyl-2-linoleoyl-GPE (16:0/18:2) levels | 0.002 | 0.005 | 0.669 |
| 5alpha-androstan-3beta,17alpha-diol disulfate levels | -0.006 | 0.005 | 0.238 |
| 5AAA,17beta-diol monosulfate (1) levels | -0.003 | 0.004 | 0.462 |
| S-methylcysteine sulfoxide levels | -0.002 | 0.006 | 0.711 |
| 1,2-dilinoleoyl-GPC (18:2/18:2) levels | 0.007 | 0.005 | 0.158 |
| 1-stearoyl-2-linoleoyl-GPE (18:0/18:2) levels | 0.006 | 0.006 | 0.340 |
| 1-myristoyl-2-arachidonoyl-GPC (14:0/20:4) levels | -0.008 | 0.005 | 0.111 |
| 1-oleoyl-2-linoleoyl-GPE (18:1/18:2) levels | 0.009 | 0.004 | 0.064 |
| N, N, N-trimethyl-5-aminovalerate levels | -4.84E-04 | 0.006 | 0.935 |
| 3-CMPFP levels | -0.008 | 0.005 | 0.099 |
| 2-naphthol sulfate levels | 0.002 | 0.006 | 0.772 |
| Pentose acid levels | 0.003 | 0.005 | 0.585 |
| 1-palmitoyl-2-linoleoyl-gpc (16:0/18:2) levels | 0.005 | 0.005 | 0.293 |
| Succinate levels | -0.007 | 0.004 | 0.060 |
| 1-methylnicotinamide levels | -0.008 | 0.007 | 0.313 |
| X-12026 levels | -0.002 | 0.005 | 0.741 |

**Abbreviations:** MR: Mendelian randomization; Se: Standard error; 3-CMPFP: 3-carboxy-4-methyl-5-pentyl-2-furanpropionate; 5AAA: 5alpha-androstan-3alpha.
